# In-hospital measurement of left ventricular ejection fraction and one-year outcomes in acute coronary syndromes: results from the IMMEDIATE Trial

**DOI:** 10.1186/s12947-016-0068-1

**Published:** 2016-08-03

**Authors:** Jayanta T. Mukherjee, Joni R. Beshansky, Robin Ruthazer, Hadeel Alkofide, Madhab Ray, David Kent, Warren J. Manning, Gordon S. Huggins, Harry P. Selker;

**Affiliations:** 1 Clinical and Translational Science Graduate Program, Sackler School of Biomedical Sciences, Tufts Clinical and Translational Science Institute, Tufts University, Boston, MA USA; 2 MCRI Center for Translational Genomics, Molecular Cardiology Research Institute, Tufts Medical Center, Boston, MA USA; 3 Center for Cardiovascular Health Services Research, Institute for Clinical Research and Health Policy Studies, Tufts Medical Center, 800 Washington St, #63, Boston, MA 02111 USA; 4 Department of Medicine (Cardiovascular Division) and Radiology, Beth Israel Deaconess Medical Center Harvard Medical School, Boston, MA USA; 5 Tufts Clinical and Translational Science Institute, Tufts University, Boston, MA USA; 6 College of Pharmacy, King Saud University, Riyadh, Saudi Arabia; 7 Regis College, Weston, MA USA; 8 Riverside Methodist Hospital, Ohio Health Heart and Vascular Physicians, Columbus, OH USA

**Keywords:** Acute coronary syndromes, Glucose-insulin-potassium, Left ventricular ejection fraction, Death, Hospitalization from heart failure

## Abstract

**Background:**

In patients with acute coronary syndrome (ACS), reduced left ventricular ejection fraction (LVEF) is a known marker for increased mortality. However, the relationship between LVEF measured during index ACS hospitalization and mortality and heart failure (HF) within 1 year are less well-defined.

**Methods:**

We performed a retrospective analysis of 445 participants in the IMMEDIATE Trial who had LVEF measured by left ventriculography or echocardiogram during hospitalization.

**Results:**

Adjusting for age and coronary artery disease (CAD) history, lower LVEF was significantly associated with 1-year mortality or hospitalization for HF. For every 5 % LVEF reduction, the hazard ratio [HR] was 1.26 (95 % CI 1.15, 1.38, *P* < 0.001). Participants with LVEF < 40 % had higher hazard of 1-year mortality or HF hospitalization than those with LVEF > 40 (HR 3.59; 95 % CI 2.05, 6.27, *P* < 0.001). The HRs for the association of LVEF with the study outcomes were similar whether measured by left ventriculography or by echocardiography, (respectively, HR 1.32; 95 % CI 1.15, 1.51 and 1.21; 95 % CI 1.106, 1.35, interaction *P* = 0.32) and whether done within 24 h or not within 24 h (respectively, HR 1.28; 95 % CI 1.10, 1.50 and 1.23; 95 % CI 1.10, 1.38, interaction *P* = 0.67).

**Conclusions:**

Among patients with ACS, lower in-hospital LVEF is associated with increased 1-year mortality or hospitalization for HF, regardless of the method or timing of the LVEF assessment. This has prognostic implications for clinical practice and suggests the possibility of using various methods of LVEF determination in clinical research.

## Background

Acute coronary syndrome (ACS) remains a leading cause of death in the United States [1]. Reduced left ventricular ejection fraction (LVEF), measured with standardized methods at 30 days following ACS, is an established marker for poor clinical outcome [2, 3], but the relationships between LVEF measured during the index ACS hospitalization and clinical outcomes are less well-defined. Moreover, LVEF measured by noninvasive transthoracic echocardiography and invasive left ventriculography has been used to risk stratify patients with ACS [4–7], but the best modality and timing of measurement of in-hospital LVEF are not known.

In the IMMEDIATE (Immediate Myocardial Metabolic Enhancement During Initial Assessment and Treatment in Emergency care) Trial [8], participants with suspected ACS who received intravenous glucose-insulin-potassium (GIK) had generally fewer serious outcomes at 1 year than those treated with placebo, but the difference did not reach statistical significance [9]. However, among those presenting with ST elevation myocardial infarction (STEMI), the composite outcomes of cardiac arrest or 1-year mortality, and of cardiac arrest, mortality, or hospitalization for heart failure (HF), were significantly reduced [9]. Long-term outcomes based on LVEF routinely measured during ACS hospitalization, in general, and in the context of GIK, have not been studied. Using data from the IMMEDIATE Trial, we sought to determine if LVEF measured during ACS hospitalization was associated with the 1-year composite outcome of all-cause mortality or hospitalization for HF.

We hypothesized that reduced in-hospital LVEF would be a marker of increased risk of death or HF hospitalization at 1 year. As a secondary goal, we tested whether in-hospital LVEF was higher among IMMEDIATE Trial participants randomized to GIK as compared to those randomized to placebo.

## Methods

### Study sample

This study analyzed a subset of data on participants enrolled in the IMMEDIATE Trial [8, 10], a randomized, placebo-controlled, double-blind clinical effectiveness trial of GIK conducted from December 2006 through July 2011, in which paramedics, aided by electrocardiograph-based decision support, enrolled 871 patients aged ≥ 30 years with high probabilities of ACS. Participants were given either GIK (30 % glucose, 50 U/L of regular insulin, and 80 mEq of KCl/L) intravenously at 1.5 mL/kg/h for 12 h, or identical-appearing placebo. This investigation included the subset of participants who had their LVEF measured during their index hospitalization by left ventriculography or echocardiography [8, 10].

### Inclusion and exclusion criteria from the IMMEDIATE Trial

Screened patients included all those transported by emergency medical services (EMS) in response to a 9-1-1 call for symptoms suggestive of ACS who were ≥ 30 years of age and had an out-of-hospital electrocardiogram (ECG) performed. Inclusion was based on paramedics’ clinical assessment of a patient likely having ACS, supplemented by decision support by the electrocardiograph-based Acute Cardiac Ischemia Time-Insensitive Predictive Instrument (ACI-TIPI) and Thrombolytic Predictive Instrument (TPI). Patients were candidates for enrollment if the ACI-TIPI prediction of ACS was 75 % or higher, if STEMI was detected by the TPI, and/or the patient met local standards for EMS identification of STEMI [10]. Patients were excluded if they had a language barrier or impaired reasoning, were prisoners or pregnant, or had clinically significant rales (Killip Class 3 or 4 HF) [8, 10].

In addition, a National Institutes of Health appointed Data Safety Monitoring Board oversaw enrollment to ensure safe and ethical study conduct throughout the trial. Informed consent was obtained from all patients in accordance with the Exception from Informed Consent Requirements for Emergency Research per the Code of Federal Regulations 21 CFR 50.24 and included community consultation, institutional review board approval from all sites, assent from patients prior to randomization, and written consent once stabilized at the hospital [11].

The Coordinating Center’s IRB at Tufts Medical Center provided approval for the overall trial along with all participating sites (University of Texas Southwestern Medical School, Dallas; Cambridge Health Alliance, Cambridge, Massachusetts; Alaska Regional Hospital, Anchorage; University of New Mexico School of Medicine, Albuquerque; Medical College of Wisconsin, Milwaukee; Medical Center of Central Georgia, Macon; Regions Hospital EMS, St. Paul, Minnesota; Avera Medical Group, Sioux Falls, South Dakota; Penn State Hershey Medical Center, Hershey, Pennsylvania; Emerson Hospital, Concord, Massachusetts; St Joseph Medical Center, Bellingham, Washington; Texas Tech University Health Sciences Center, El Paso; Yale New Haven Hospital, Connecticut)

### Data collection

Data were collected by trained study staff. They were instructed to record LVEF as measured by cardiac catheterization; if the catheterization was not done, and LVEF was available by echocardiogram, this was recorded. The dates and times of echocardiograms were not routinely recorded, so medical records were reviewed to obtain them where possible. Based on emergency department (ED) presentation date and dates of LVEF measurement by left ventriculography or echocardiography, we classified a participant’s LVEF as “early” (within 24 h of ED presentation), or “not early” if measured more than 24 h after ED presentation. We could not find the date and time of LVEF measurement for 94 out of 445 participants; for this study we assigned them to the not early group, as the majority (92) had echocardiograms, and our search showed that the majority of the echocardiograms were done more than 24 h after ED presentation.

### Data analysis

Statistical analyses were performed using R, version 2.15.2. Tests were two-sided, using alpha ≤ 0.05 as statistical significance. Comparison of means of in-hospital LVEF between the GIK and placebo groups, between early and not early LVEF measurement, and between catheterization and echocardiogram measurement all used two-sample student t-tests. Just for the Kaplan–Meier survival curves, they were plotted for participants with LVEFs in normal (55–70 %), mildly abnormal (40–54 %), moderately abnormal (25–39 %) and severely abnormal (<25 %) categories. Cox proportional hazards models were used to estimate univariate hazard ratios (HRs) for LVEF associated with the composite outcome of death or hospitalization for HF. Based on clinical and statistical significance in those analyses, we chose candidate variables for possible inclusion in a multivariable model to estimate adjusted HRs for the composite outcome of death or hospitalization for HF at 1 year. Using the final adjusted model, we also tested for dichotomous variables of timing of LVEF (early vs. not early) and the measurement method (catheterization vs. echocardiogram). Two-way interactions between LVEF and timing (early vs. not early) were tested in the Cox proportional hazard model of the composite outcome, adjusting for age and coronary artery disease (CAD). A similar analysis was done to test the two-way interaction between LVEF and test method (catheterization vs. echocardiogram). We checked assumptions needed for proportional hazards analyses by plotting and testing Schoenfeld residuals as related to time.

## Results

### Baseline demographics

Table 1 shows demographic and clinical features of participants having the composite outcome of death or hospitalization for HF at 1 year (*n* = 52) and those who did not (*n* = 393). Participants with the composite outcomes were older and more frequently had medical histories of CAD, HF, diabetes, hypertension, stroke, and hyperlipidemia. Rates of acute myocardial infarction were similar in both groups, but those participants that had the composite outcome presented with a significantly higher Killip Class. Please see Appendices below.

**Table 1:** Baseline characteristics of participants with LVEF and outcomes (*N* = 445)

Variables	Participants with neither death nor hospitalization for HF at 1 year *N* = 393	Participants with death or hospitalization for HF at 1 year *N* = 52
Age (mean ± SD, years)	61 ± 12.1 (393)	71 ± 13.0 (52)
Gender, % Male	75.6 (297)	69.2 (36)
White race (vs. non- White race)	86.7 % (341)	88.5 % (42)
Body Mass Index, (mean ± SD, units)	28.9 ± 6.3 (372)	27.9 ± 6.0 (42)
Time from onset of symptoms to treatment (minutes)		
Mean ± SD,	146.8 ± 206.4 (353)	111.7 ± 109.3 (37)
median < IQR >	78 < 50.0–140.0 >	65 < 40.0–145.0 >
Chief Complaint on Presentation		
Chest Pain	91.8 % (361)	84.6 % (44)
Medical History, % (n)		
CAD (MI, PCI or CABG)	30.8 % (121)	69.2 % (36)
Heart Failure	6.7 % (25)	23.1 % (12)
Diabetes Mellitus	21.1 % (83)	34.7 % (18)
Hypertension	62.3 % (245)	78.8 % (41)
Stroke	5.9 % (23)	15.4 % (8)
Hyperlipidemia	46.8 % (184)	65.4 % (34)
Hospital Reperfusion Treatment, %(n)		
PCI	73.8 % (290)	59.6 % (31)
Thrombolytic therapy	0.8 % (3)	9.6 % (5)
CABG	5.1 % (20)	7.7 % (4)
Confirmed Diagnosis, % (n)		
Acute Myocardial Infarction	80.4 % (316)	78.8 % (41)
Killip Class		
1	76.1 % (299)	51.9 % (27)
2	1.8 % (7)	1.3 % (7)
3	0.5 % (2)	3.8 % (2)
4	2.0 % (8)	9.6 % (5)
Unstable Angina	12.7 % (50)	9.6 % (5)
ECG based findings		
ST Elevation on Presentation (ECG)	70.8 % (264)	66.0 % (33)
ACI-TIPI score, (Mean ± SD)	80.7 ± 17.6 (386)	84.8 ± 11.2 (49)
LVEF in %, Median (IQR)	50 (40,60)	35 (25,45)
GIK (vs. Placebo)	47.6 % (187)	38.5 % (20)

Data on LVEF measured by left ventriculography or echocardiography during the index hospitalization were available for 445 of 871 study participants. There were significant differences in baseline demographics and clinical characteristics of participants with and without in-hospital LVEFs recorded. Of participants with in-hospital LVEF measured, 92.6 % had confirmed diagnoses of ACS, compared with 33.1 % among those without measured LVEF. There were no significant differences between characteristics of participants who had in-hospital LVEF recorded by left ventriculography vs. echocardiography. For 248 participants, LVEF was measured early (219 [80.1 %] by catheterization), defined as measurement within 24 h of presentation, and for 197 participants, the LVEF measurement was performed not early (54 [19.7 %] by left ventriculography).

### Univariate models of LVEF and outcomes

**Fig. 1:**
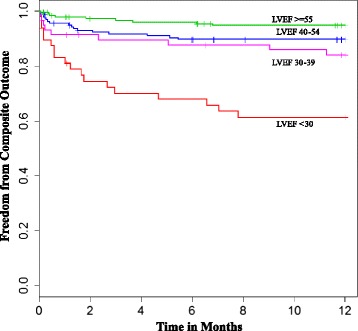
Kaplan–Meier plot for categories of LVEF

Participants with the lowest LVEF had the lowest survival and the greatest incidence of composite outcomes (Fig. 1). Univariate Cox proportional hazard models were used to identify factors associated with the composite outcome (Table 2). A reduction of LVEF by 5 % decrements was significantly associated with higher hazards of death or hospitalization for HF over 1 year (HR = 1.31, 95 % CI: 1.20–1.45, *P* < 0.001). Also significant was age, expressed in 10-year increments (HR = 1.79, 95 % CI: 1.44–2.24, *P* < 0.001), prior CAD (HR = 4.46, 95 % CI: 2.47–8.04, *P* < 0.001), HF (HR = 3.56, 95 % CI: 1.86–6.79, *P* < 0.001), diabetes (HR = 1.88, 95 % CI: 1.06–3.34, *P* = 0.030), hypertension (HR = 2.17, 95 % CI: 1.12–4.23, *P* = 0.022), and stroke (HR = 2.56, 95 % CI: 1.20–5.43, *P* = 0.015).

**Table 2:** Unadjusted and multivariable-adjusted Cox proportionate hazard models for composite outcomes (death and hospitalization for HF) by LVEF measured in-hospital (*N* = 445, no of events = 52)

Unadjusted cox proportional hazard model		
Variables	HR (95 % CI)	*P*-value
LVEF/5 % decrease	1.31 (1.20,1.45)	< 0.001
Age/10 years increase	1.79 (1.44, 2.24)	< 0.001
Gender, Male	1.37 (0.41, 1.32)	0.23
White race (nonwhite race reference)	1.13 (0.48, 2.65)	0.77
Body Mass Index	0.97 (0.92, 1.02)	0.32
Time from onset of symptoms to treatment*	0.98 (0.99, 1.00)	0.33
Chief complaint on presentation		
Chest pain	0.53 (0.23, 1.12)	0.10
Out of Hospital ECG ST-Elevation	0.84 (0.47, 1.50)	0.55
ACI-TIPI Score	1.02 (0.99, 1.04)	0.12
Medical history		
CAD (MI, PCI or CABG)	4.46 (2.47, 8.04)	< 0.001
Heart Failure	3.56 (1.86, 6.79)	< 0.001
Diabetes	1.88 (1.06, 3.34)	0.03
Hypertension	2.17 (1.12, 4.23)	0.02
Stroke	2.56 (1.20, 5.43)	0.02
Multivariable adjusted cox proportional hazard models		
Variables	HR (95 % CI)	*P* value
Outcome (Composite)		
LVEF/5 % lower (combined)	1.26 (1.15, 1.38)	< 0.001
Age/10 years Increase	1.73 (1.38, 2.18)	< 0.001
History of CAD (MI, PCI, or CABG)	2.97 (1.62, 5.43)	< 0.001
Outcome (Death)		
LVEF/5 % lower (combined)	1.18 (1.06, 1.32)	0.002
Age/10 years Increase	1.94 (1.47, 2.55)	<0.001
History of CAD (MI, PCI, or CABG)	3.56 (1.69, 7.49)	< 0.001
Outcome (Hospitalization for HF)		
LVEF/5 % lower (combined)	1.37 (1.18, 1.58)	< 0.001
Age/10 years Increase	1.35 (0.95, 1.91)	0.09
History of CAD (MI, PCI, or CABG)	2.27 (0.93, 5.56)	0.007

### Multivariable models of LVEF and outcomes

Multivariate Cox models were used to better characterize the association of LVEF with the composite outcome. In stepwise regression, candidate parameters for inclusion included LVEF expressed by 5 % decrements; age, sex, history of CAD, HF and diabetes. The model that had the lowest akaike information criteria (best fit) following the selection process included only LVEF, age, and CAD. The C statistic (which is equivalent to the receiver-operating characteristic (ROC) curve area) for that model is 0.805 (SE = 0.04).

The effect of 5 % lower LVEF on 1-year death or hospitalization for HF remained statistically significant after adjusting for age and history of CAD (HR = 1.26; 95 % CI: 1.15–1.38, *P* < 0.001) when LVEF was used as a continuous variable. When LVEF was tested as a binary variable and dichotomized, participants with LVEF < 40 % had higher hazard of 1-year mortality and hospitalization for HF compared to participants with LVEF ≥ 40 %, after adjusting for age and history of CAD (HR = 3.59; 95 % CI: 2.05–6.27, *P* < 0.001). The proportional hazard assumption was not violated. Separating the composite outcome into its components, reductions of LVEF by 5 % decrements were also significantly associated with higher hazard of death and hospitalization for HF when adjusted for age and history of CAD (HR = 1.18; 95 % CI: 1.06–1.32, *P* = 0.002; and HR = 1.37; 95 % CI: 1.18–1.58, *P* < 0.001, respectively) (Table 2).

The HRs for the association of LVEF with the study outcomes were similar whether measured by left ventriculography or by echocardiography, (respectively, HR = 1.32; 95 % CI 1.15, 1.51 and 1.21; 95 % CI 1.106, 1.35) and whether done within 24 h or not within 24 h (respectively, HR = 1.28; 95 % CI 1.10, 1.50 and 1.23; 95 % CI 1.10, 1.38). Tests for interactions of LVEF and measurement method and LVEF and timing did not reach significance (Table 3). Controlling for method of LVEF measurement (left ventriculography vs. echocardiography) or timing of measurement (early vs. not early) did not alter the association of LVEF with the outcome (Table 4). Additionally, while having an echocardiogram (vs. left ventriculography) and being not early (vs. early) were associated with the 1-year outcome, neither was statistically significant.

**Table 3:** Association of LVEF with composite outcome, stratified by timing and type of procedure

Variable	Adjusted HR (95 % CI)	Interaction *P* value
LVEF- by catheterization/5 % lower^a^	1.32 (1.15, 1.51)	0.32
LVEF- by echocardiogram/5 % lower^a^	1.20 (1.06, 1.35)	
LVEF- early/5 % lower^a^	1.28 (1.10, 1.50)	0.67
LVEF- not early/5 % lower*	1.23 (1.10, 1.38)	

^a^Adjusted for age and history of CAD

**Table 4:** Model for LVEF measured by cardiac catheterization (reference echocardiogram) and not early (reference Early) (*N* = 445, no of events = 52)

Variable	HR (95 % CI)	*P* value
LVEF/5 % lower (combined)	1.25 (1.14, 1.37)	< 0.001
Age/10 years Increase	1.71 (1.36, 2.15)	< 0.001
History of CAD (MI, PCI, or CABG)	2.94 (1.60, 5.40)	< 0.001
LVEF - echocardiogram (reference catheterization)	1.62 (0.93, 2.80)	0.08
LVEF/5 % lower (combined)	1.25 (1.13, 1.36)	< 0.001
Age/10 years Increase	1.71 (1.36, 2.15)	< 0.001
History of CAD (MI, PCI, or CABG)	2.86 (1.56, 5.26)	< 0.001
LVEF - not early (reference early)	1.53 (0.86, 2.69)	0.14

**Table 5:** Distribution of LVEF % by experimental treatment groups

Variable	GIK, Mean (SD) Median LVEF%, (*N*)	Placebo Mean (SD) Median LVEF%, (*N*)	*P* value for Mean
LVEF (Catheterization and Echocardiogram)	48.6 (14) 50 (207)	46.2 (14) 45 (238)	0.07
LVEF (Catheterization)	49.3 (14) 50 (120)	48.3 (14) 50 (153)	0.33
LVEF (Echocardiogram)	47.6 (14) 50 (87)	43.6 (14) 45 (85)	0.08
LVEF (Early^a^)	49.2 (13) 50 (110)	47.4 (13) 50 (138)	0.27
LVEF (Not early^b^)	47.8 (15) 50 (97)	44.6 (15) 45 (100)	0.13

^a^Early when the LVEF was measured within 0 days of ED arrival

^b^Not early when LVEF was measured 1 or more days after ED arrival or with unknown dates

Sensitivity analyses were performed in which we re-estimated HRs for LVEF, adjusting not only for already specified covariates age and CAD history, but also for sex, HF history, and diabetes. Reductions of LVEF by 5 % were significantly associated with higher hazard of the 1-year composite outcome (HR = 1.25; 95 % CI: 1.14–1.38, *P* < 0.001). Also, the HRs for the association of LVEF with the study outcome when measured by left ventriculography vs. by echocardiography, and for when measured early vs. not early, were similar. Finally, we studied the association of the IMMEDIATE Trial treatment, GIK vs. placebo, on in-hospital LVEF, stratified by method (catheterization vs. echocardiogram) and timing (early vs. not early. There was a trend toward higher in-hospital LVEF among those receiving GIK. When measured by echocardiography alone, median LVEF with GIK 50 % vs 45 % with placebo (*P* = 0.08). When measured by either echocardiography or catheterization, the median LVEF with GIK was 50 % vs 45 % with placebo (P–0.07) (Table 5).

## Discussion

In this post hoc analysis of IMMEDIATE trial participants who had in-hospital LVEF assessment we demonstrate that in-hospital LVEF for patients with ACS can identify patients at risk for death or HF hospitalization within 1 year. Our results do not favor left ventriculography or echocardiography as a preferred modality for LVEF measurement; as the predictive ability of LVEF was similar for both modalities. These results also do not support a need for LVEF assessment within 24 h of presentation. These results have practical importance for the care of patients with ACS because our data suggest that assessment of LVEF measured at any point in the hospitalization can be used to predict patients at risk of death or hospitalization for HF within 1 year.

In-hospital LVEF assessment is not routinely performed in ACS patients. One study demonstrated over 40.8 % of patients with non-ST elevation myocardial infarction did not have an LVEF assessment [12, 13], and the percentage of patients getting LVEF measurement has steadily increased in the last two decades [14, 15]. LVEF is superior to end systolic volume index and infarct size in predicting 6-month mortality after myocardial infarction [2]. The presence of LV dysfunction on baseline left ventriculography in patients enrolled in the HORIZONS–AMI trial who underwent primary PCI was a powerful predictor of early and late mortality irrespective of the extent of coronary artery disease [7]. The age, creatinine and ejection fraction score (ACEF), as proposed by J H Lee et al. may be used to stratify the 1-year mortality risk in 30-day survivors who underwent percutaneous coronary intervention (PCI) after acute myocardial infarction [16]. In spite of optimal medical therapy and clinically driven percutaneous coronary intervention (PCI), LVEF and angiographic burden of disease at baseline retain prognostic importance for patients aggressively treated for stable CAD [17].

Our study, which featured the full spectrum of ACS presentations, suggests that useful information can be obtained by measuring LVEF by either left ventriculography or echocardiography at any time prior to hospital discharge. These findings have important implications for clinical effectiveness research, because we demonstrate the utility of collecting data from clinically-indicated and performed LVEF assessments in large multicenter clinical trials as a means to define the risk of longitudinal cardiac outcomes. If substantiated as a research tool, this finding has the potential to reduce the need for core lab LVEF assessments, which can be quite costly.

In the IMMEDIATE Trial, GIK did not significantly reduce the primary endpoint of progression of unstable angina to AMI, but it did significantly reduce the composite endpoint of cardiac arrest or mortality, and in the biological mechanism cohort, the median infarct size measured by sestamibi SPECT imaging at 30 days was 80 % lower in the GIK compared to the placebo group. Further, there was a trend for better LVEF measured by SPECT imaging at 30 days with GIK [8]. Our study also showed that GIK had a trend toward improved in-hospital LVEF when compared to placebo especially when measured by echocardiography. Future well-powered studies will be required to further delineate the effect of GIK on infarct size and long-term left ventricular function.

Our study has several limitations. It is a retrospective analysis using data from a prospective randomized controlled trial on a subset with 445 participants who had LVEF measured by echocardiogram or cardiac catheterization. While the sample is not large, we had 52 composite outcomes in the group. The study cohort includes patients with high-risk ACS. The LVEF was measured by two different modalities at slightly different times, which could introduce bias since the LVEF measured by left ventriculography was during revascularization and LVEF measured by echocardiography was primarily obtained prior to hospital discharge. However, our study showed that the methodology and the timing of the LVEF measurement did not matter. The study cannot compare the in-hospital LVEF measurement with an assessment made by a core lab, and hence we cannot say that clinical LVEF results necessarily give the same information as measurements made by a core laboratory. However, we did find that LVEF measured in the hospital and not by a core laboratory was significantly associated with 1-year outcomes of death and HF hospitalization.

## Conclusion

Lower in-hospital LVEF is associated with higher rates of 1-year mortality and hospitalization from HF in patients hospitalized with ACS, regardless of the method or timing of LVEF assessment. This has prognostic implications for clinical practice and suggests the possibility of using various methods of LVEF determination in clinical research.

## Abbreviations

ACI-TIPI, Acute Cardiac Ischemia Time-Insensitive Predictive Instrument; ACS, acute coronary syndrome; CAD, coronary artery disease; ED, emergency department; GIK, glucose-insulin-potassium; HR, hazard ratio; HF, heart failure; LVEF, left ventricular ejection fraction; STEMI, ST elevation myocardial infarction; TPI, Thrombolytic Predictive Instrument.

## Appendices

### A. Baseline characteristics

Figure 1. Participants with LVEF and outcomes

Table 1. Baseline characteristics of participants with and without LVEF

Table 2. Baseline characteristics of participants with LVEF measured by catheterization or echocardiogram

Table 3. Baseline characteristics of participants with LVEF measured early or not early

Table 4. Difference in days by catheterization and echocardiogram

### B. Intermediate models

Table 1. Composite outcome and LVEF measured regardless of modality and timing

Table 2. Composite outcome and LVEF measured by catheterization and echocardiogram

Table 3. Composite outcome and LVEF measured early and not early

### C. Sensitivity analyses

Figure 1. Kaplan-Meir survival curve for LVEF < 40 % & ≥ 40 %

Table 1. Composite outcome in participants with LVEF < 40 % & ≥ 40 %

Table 2. Composite outcome and LVEF measured in participants who had PCI

Table 3. Composite outcome and LVEF measured in participants who had confirmed MI
